# A bidirectional link between metabolic syndrome and elevation in alanine aminotransferase in elderly female: a longitudinal community study

**DOI:** 10.3389/fcvm.2023.1156123

**Published:** 2023-06-20

**Authors:** Na Wu, Mofan Feng, Hanhua Zhao, Nan Tang, Yalan Xiong, Xinyu Shi, Dong Li, Hualing Song, Shengfu You, Jianying Wang, Lei Zhang, Guang Ji, Baocheng Liu

**Affiliations:** ^1^ Shanghai Innovation Center of Traditional Chinese Medicine Health Service, School of Public Health, Shanghai University of Traditional Chinese Medicine, Shanghai, China; ^2^ Bio-X Institutes, Key Laboratory for the Genetics of Developmental and Neuropsychiatric Disorders, Shanghai Jiao Tong University, Shanghai, China; ^3^ Department of Sport Science, College of Education, Zhejiang University, Hangzhou, China; ^4^ Zhangjiang Community Health Service Center of Pudong New District, Shanghai, China; ^5^ Institute of Digestive Diseases, Longhua Hospital, Shanghai University of Traditional Chinese Medicine, Shanghai, China

**Keywords:** metabolic syndrome, alanine aminotransferase, bidirectional relation, elderly population, longitudinal study

## Abstract

Pre-obesity, as a significant risk factor for the progression of metabolic syndrome (MS), has become a prevalent public health threat globally. In this three-year longitudinal study of pre-obese women at baseline, the goal was to clarify the female-specific bidirectional relationship between the risk of MS and blood alanine aminotransferase. In this manuscript, the MS score was determined using the following equation: MS score = 2*waist/height + fasting glucose/5.6 + TG/1.7 + SBP/130—HDL/1.02 for men and 1.28 for women, which is highly related to the risk of MS. With 2,338 participants, a hierarchical nonlinear model with random effects was utilized to analyze the temporal trends of serum characteristics from 2017 to 2019. A bivariate cross-lagged panel model (CLPM) was employed to estimate the structural relations of frequently measured variables at three different time points to determine the directionality of the relationship between the risk of MS and serum characteristics. MassARRAY Analyzer 4 platforms were used to evaluate and genotype candidate SNPs. In this study, the MS score only rose with age in females; it was positively correlated with serum alanine aminotransferase (ALT) in females; the CLPM revealed that the MS score in 2017 predicted ALT in 2018 (β = 0.066, *p* < 0.001); and ALT in 2018 predicted an MS score in 2019 (β = 0.037, *p* < 0.050); both relationships were seen in females. Additionally, the MS score in elderly females with NAFLD was related to the rs295 in the lipoprotein lipase (*LPL*) gene (*p* = 0.042). Our work showed that there may be female-specific causal correlations between elevated ALT and risk of MS and that the polymorphism rs295 in *LPL* may serve as a marker for the prognosis of MS. The genetic roles of rs295 in the *LPL* gene in the onset of MS and the development of ALT in the elderly Chinese Han population are thus provided by this, offering one potential mechanism.

## Introduction

By 2035, 25% of the population will be aged 60 years and over in China (1); ageing has become one of the major forces driving the global epidemic of chronic diseases. Pre-obesity, as a significant risk factor for the progression of several metabolic syndromes (MS)-related chronic conditions, including hypertension, dyslipidemia, type 2 diabetes (T2D), and non-alcoholic fatty liver diseases (NAFLD) (2), has been getting increased attention. Pre-obesity, also called overweight, is a prevalent public health threat globally, including in China, where pre-obesity prevalence was 34.3% in adults in 2019 (3).

Traditionally, body mass index (BMI) is widely used to classify overweight and obesity (4). However, waist circumference (WC) measurement seems more accurate in determining central obesity, and it has been suggested to be associated with MS (5). Notably, MS often co-exists with NAFLD (6, 7) including simple steatosis and non-alcoholic steatohepatitis (NASH), which would deteriorate into fibrosis and cirrhosis (8). Subjects with NAFLD usually have an abnormal level of alanine aminotransferase (ALT) and aspartate aminotransferase (AST) (9). Among them, ALT is an enzyme that involves the process of amination between glutamic acid and oxaloacetate; it is found mostly among cytosol of hepatocytes, and serum ALT increased accompanied by apoptosis and injury of hepatocytes (10), that's why it is used as an indicator of liver function to reflect the hepatic inflammation in patients with diverse liver diseases, including NAFLD. According to the previous research finding, elevated ALT level was strongly associated with an increased risk of NAFLD (11). While some other studies observed that NAFLD patients diagnosed by histology presented the normal ALT value (12, 13); furthermore, there was still a normal ALT value in the patients with hepatocellular carcinoma (14). A notable risk factor for cardiometabolic diseases is hemoglobin, the circulatory system's primary oxygen carrier. In a 20-year follow-up research, it was found that increased hemoglobin had a negative relationship with metabolic profile (15), and a causal analysis suggested that even lower hemoglobin levels may be beneficial for metabolic health (16).

It was reported that ALT was related to MS-related variables, e.g., BMI, WC, and triglyceride (TG) in the population of the United States (17); and several studies have reported that ALT could predict type 2 diabetes (T2D) using regression analysis (18, 19). Based on these findings, it is of interest to elucidate the role of ALT in the occurrence and development of MS. However, whether NAFLD-associated biomarkers are longitudinally associated with the development of MS has not been explored in the elderly Chinese population. In addition, MS, a multifactorial chronic disease, is induced by the interaction between genetic predisposition and environment factors (20). In contrast, genetic polymorphisms related to aging with MS have been reported less.

The aim of this study was to evaluate the longitudinal associations of liver markers, e.g., ALT, with the odds of MS and to explore the association of genetic polymorphisms with the odds of MS among participants in the elderly female Chinese population who were known to be pre-obese at baseline.

## Methods and materials

### Subjects

**Figure 1:**
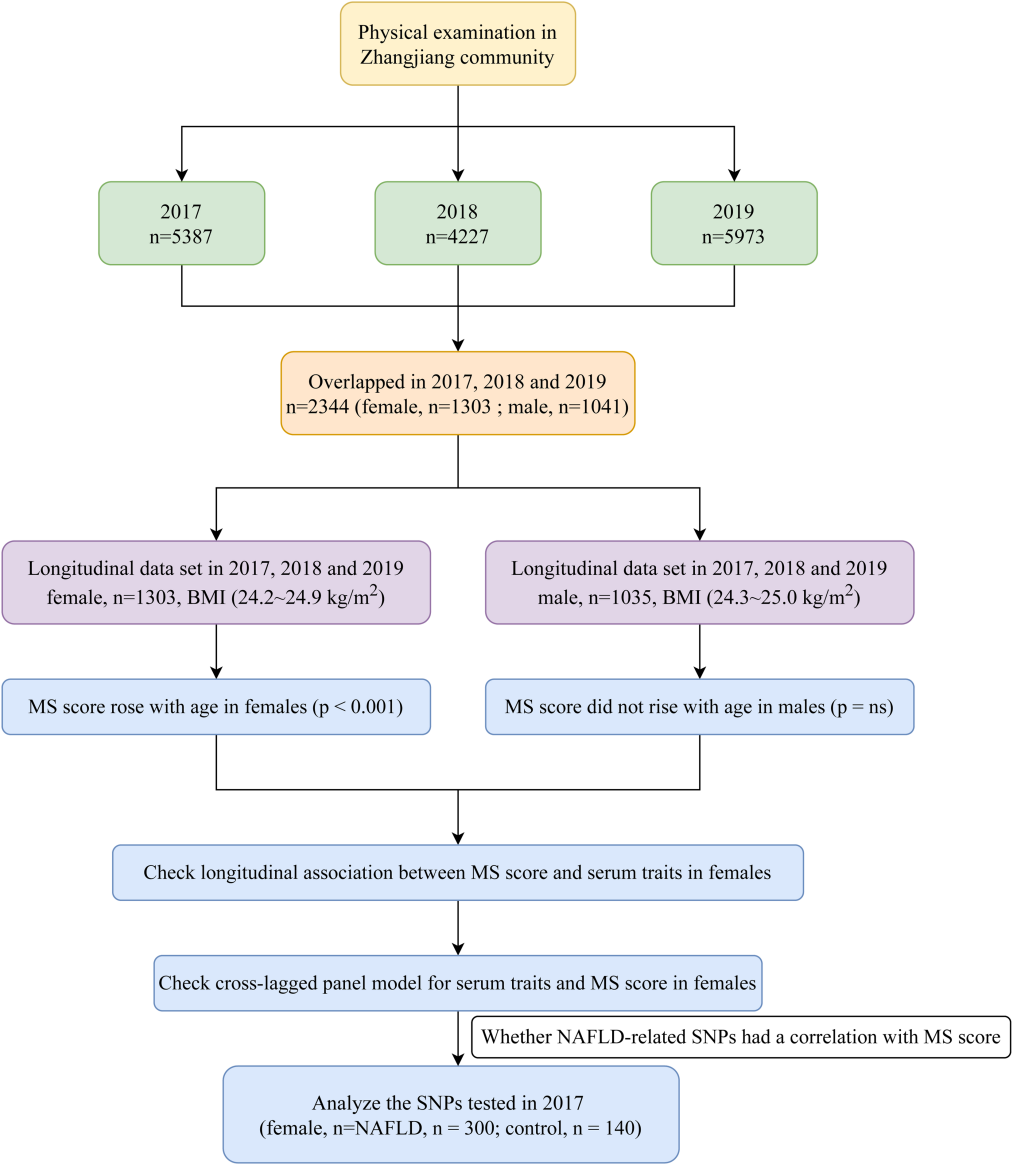
Study flowchart.

The Zhangjiang neighborhood of Shanghai's Pudong District Health Care Service Centers, where annual health checks were conducted from 2017 to 2019, served as the recruitment pool for the study's outpatient participants. The Helsinki Declaration was followed throughout the study. The Shanghai Innovation Center of Traditional Chinese Medicine Health Service has created a standard protocol, and the Shanghai University of Traditional Chinese Medicine Ethics Committee has given it the go light. All subjects provided their consent. Measurements can be completed by participants over 60 who reside in Shanghai, and informed consent was a requirement for inclusion. Participants with mental illnesses, cancerous tumors, or inadequate medical records were not included in this study. Six male individuals under 60 were eliminated from the study, leaving 2,338 (1,303 female; 1,035 male) senior Chinese subjects with complete data for the years 2017, 2018, and 2019 (Figure 1). As the MS score rose with age only in females (*p* < 0.001) but did not change significantly with age in males (data was shown in the results section), the focus of our future analysis was primarily on female MS. Additionally, we also conducted a sub-analysis of SNPs in 2017 to clarify the relationship between genetic variations and MS score to check whether NAFLD-related SNPs correlated with MS score in the elderly female Chinese Han population.

### Anthropometry and physical examinations

Weight (kg) divided by height squared is how the body mass index (BMI) is computed (m^2^). Using electronic sphygmomanometers, blood pressure was measured (Bio-space, Cheonan, South Korea). The experienced specialist accurately measured waist circumferences with a non-stretch tape. After overnight fasting, blood samples were taken from the antecubital vein in the morning. The biochemistry analyzer was used to assess fasting glucose, alanine transaminase (ALT), aspartate transaminase (AST), total cholesterol (TC), low-density lipoprotein (LDL), high-density lipoprotein (HDL), TG, hemoglobin, and urea (Hitachi, Tokyo, Japan).

### Genotyping

The EZ1 DNA Blood 350 L kit (Qiagen) was used to extract genomic DNA from venous blood leukocytes in accordance with the manufacturer's instructions for genotyping. Six SNPs were genotyped using a matrix-assisted laser desorption/ionization time-off light mass spectrometer in the MassARRAY Analyzer 4 platforms, including rs925946 in brain-derived neurotrophic factor (*BDNF*), rs15285, rs295, and rs301 in lipoprotein lipase (*LPL*), and rs7901695 and rs7903146 in the transcription factor 7 like 2 (*TCF7L2*), which were identified potential risk loci in metabolic syndrome (21–23). With the online Assay Design Suite 2.0 software, primers, and probes were chosen. The manufacturers' instructions for the polymerase chain reaction were followed. Further specific details regarding the settings for polymerase chain reactions and primers are provided upon request.

### Statistical analysis

Using IBM SPSS Statistics, the Shapiro-Wilk test was applied to determine whether the data were normal (version 26.0). Natural logarithms of the data were employed if they were not normally distributed. The mean and standard deviation for clinical data were displayed. Percentages were determined for categorical data. MS score was determined using the following equation: MS score = 2*waist/height + fasting glucose/5.6 + TG/1.7 + SBP/130—HDL/1.02 for men and 1.28 for women (24).

The temporal trends of anthropometric and serum characteristics from 2017 to 2019 were evaluated using a hierarchical nonlinear model with random effects (MLwin 2.26 software, Multiple Project, Institute of Education, University of London, UK). In order to explain how the target variables changed over time, age was entered as the explanatory variable in the form of polynomial spline functions. This hierarchical model analysis was also applied with ALT, AST, urea, and hemoglobin as independent variables and MS score as an outcome variable to ascertain the longitudinal relationships of the MS score with serum characteristics.

A bivariate cross-lagged panel model (CLPM) was employed to estimate the structural relations of frequently measured variables at three different time points in order to evaluate the directional relationship between MS score and ALT/hemoglobin in females from 2017 to 2019. The auto-regressive component of the model shows how the variables are temporally stable from one time point to the next. The MS score and ALT in females during the follow-up were examined as reciprocal relationships between the variables at subsequent time points using CLPM. Lavaan used R software for structural equation modeling (25).

The Hardy-Weinberg equilibrium (HWE) and genotypic and allelic distributions were examined for the sub-analysis using the online tool SHEsis (http://analysis.bio-x.cn/myAnalysis.php) (14). The “SNPassoc” R package was used to investigate the relationships between each SNP and MS score/ALT in five genetic models (codominant, dominant, recessive, over-dominant, and log-additive models, respectively) (26). Statistics were judged significant at *p* < 0.05.

## Results

### Patterns of longitudinal anthropometric characteristic change during a three-year period

Table 1 presents the characteristics of the study participants. In females, BMI in 2017 (24.20 kg/m^2^) was much lower than it in 2018 (24.66 kg/m^2^) and 2019 (24.91 kg/m^2^) (p = 0.001, *p* < 0.001); DBP, ALT, AST, TC, and LDL in 2017 were much higher than them in 2018 and 2019 (*p* < 0.001, *p* < 0.001; *p* < 0.001, *p* = 0.020; *p* = 0.006, *p* = 0.011; *p* < 0.001, *p* < 0.001; *p* < 0.001, *p* < 0.001); WC in 2017 (81.68 cm) was much lower than it in 2018 (82.55 cm) (*p* = 0.010), so did urea and TG (*p* = 0.040; *p* = 0.002). Similar change patterns were also found in males.

**Table 1:** Participants information of this longitudinal study.

Female (*N* = 1303)							Male (*N* = 1035)					
	2017	2018	2019	P1	P2	P3	2017	2018	2019	P1	P2	P3
	Mean ± SD	Mean ± SD	Mean ± SD				Mean ± SD	Mean ± SD	Mean ± SD			
Age (years)	71 ± 5.67	72 ± 5.62	73 ± 5.62				71 ± 5.67	72 ± 5.62	73 ± 5.62			
BMI (kg/m^2^)	24.2 ± 3.49	24.66 ± 3.46	24.91 ± 3.49	0.001	<0.001	0.062	24.32 ± 3.19	24.87 ± 3.29	25.03 ± 3.21	<0.001	<0.001	0.286
SBP (mmHg)	144 ± 21.74	142 ± 21.26	144 ± 19.85	0.082	0.875	0.113	142 ± 21.24	140 ± 19.96	140 ± 18.69	0.039	0.033	0.094
DBP (mmHg)	82 ± 11.22	76 ± 9.45	77 ± 7.41	<0.001	<0.001	<0.001	81 ± 11.54	76 ± 9.27	78 ± 7.85	<0.001	<0.001	0.001
WC (cm)	81.68 ± 8.84	82.55 ± 8.79	81.52 ± 8.64	0.011	0.633	0.003	84.99 ± 9.18	85.56 ± 8.91	84.3 ± 8.66	0.145	0.074	0.001
ALT (U/L)	22.97 ± 13.79	19.89 ± 12.18	21.26 ± 26.67	<0.001	0.020	0.061	24.58 ± 13.48	21.74 ± 14.68	22.28 ± 13.12	<0.001	<0.001	0.378
AST (U/L)	23.73 ± 8.74	22.39 ± 9.96	22.49 ± 16.92	0.006	0.011	0.826	23.32 ± 7.85	22.24 ± 11.55	21.64 ± 7.17	0.007	<0.001	0.128
Urea (mmol/L)	5.52 ± 1.51	5.64 ± 1.44	5.57 ± 1.48	0.040	0.360	0.253	5.58 ± 1.53	5.76 ± 1.59	5.65 ± 1.65	0.010	0.280	0.136
Glucose (mmol/L)	6.09 ± 1.52	6.05 ± 1.68	6.07 ± 1.71	0.573	0.784	0.773	6.2 ± 1.64	6.15 ± 1.71	6.11 ± 1.69	0.518	0.215	0.552
Hemoglobin (g/L)	132.82 ± 10.28	132.81 ± 10.85	133.84 ± 11.81	0.980	0.017	0.016	147.83 ± 12.32	146.9 ± 12.85	146.84 ± 13.63	0.099	0.079	0.913
TC (mmol/L)	5.27 ± 0.93	5.02 ± 0.89	4.95 ± 0.94	<0.001	<0.001	0.072	4.79 ± 0.91	4.52 ± 0.97	4.47 ± 0.87	<0.001	<0.001	0.205
HDL (mmol/L)	1.3 ± 0.28	1.3 ± 0.28	1.32 ± 0.3	0.697	0.166	0.076	1.18 ± 0.25	1.17 ± 0.24	1.19 ± 0.27	0.256	0.280	0.027
LDL (mmol/L)	3.25 ± 0.83	3.12 ± 0.78	1.32 ± 0.3	<0.001	<0.001	<0.001	3.03 ± 0.85	2.88 ± 0.79	1.19 ± 0.27	<0.001	<0.001	<0.001
TG (mmol/L)	1.53 ± 1.03	1.68 ± 1.24	1.63 ± 1.18	0.002	0.034	0.293	1.36 ± 1.03	1.52 ± 1.46	1.43 ± 1.07	0.002	0.150	0.108

ALT, alanine aminotransferase; AST, aspartate aminotransferase; BMI, body mass index; DBP, diastolic blood pressure; HDL, high density lipoprotein; LDL, low density lipoprotein; SBP, systolic blood pressure; TC, total cholesterol; TG, triglyceride; WC, waist circumference. P1 indicates p value between 2017 and 2018, P2 indicates p value between 2018 and 2019, and P3 indicates p value between 2017 and 2019.

**Figure 2:**
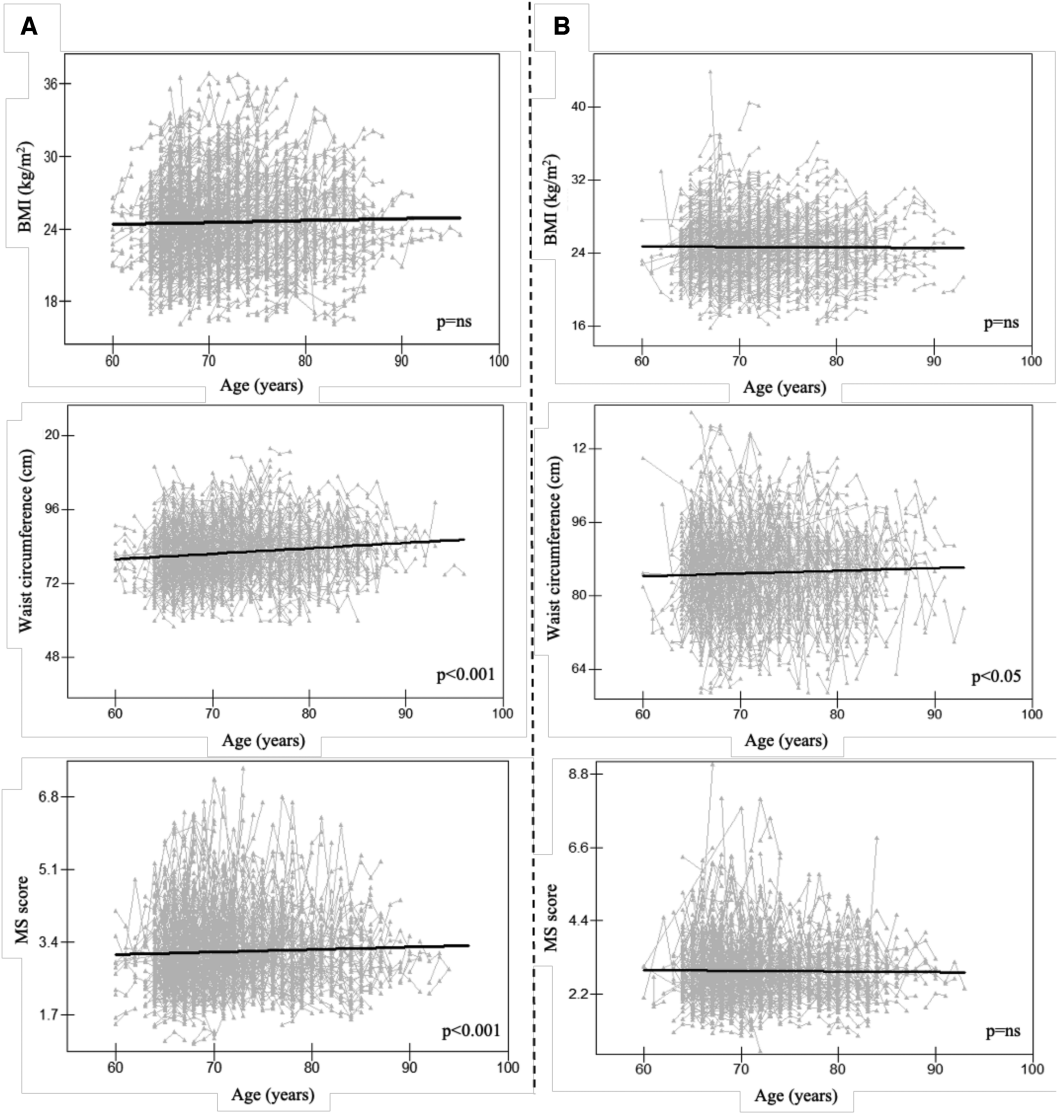
Longitudinal change pattern in females (**A**) and males (**B**), Alt text: BMI, body mass index; MS score, metabolic syndrome score.

WC increased significantly with age in both males and females (*p* < 0.001, *p* < 0.050, Figure 2) despite BMI showing no significant trend. It is noteworthy that the MS score rose with age in females (*p* < 0.001) but did not change significantly with age in males. As a result, the focus of our future analysis was primarily on female MS (Figure 1).

### Females' serum characteristics and MS score throughout time

**Figure 3:**
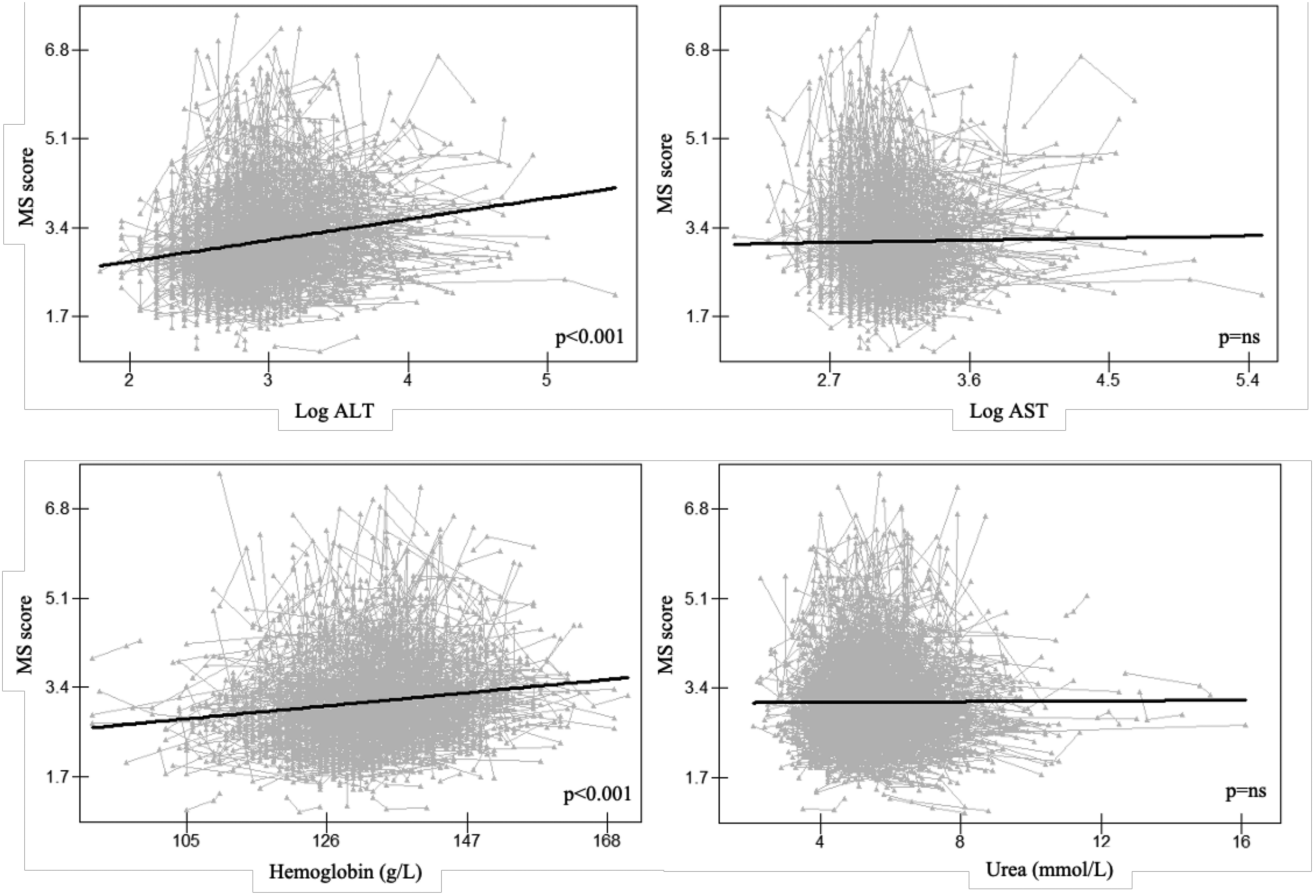
Longitudinal association between MS score and serum traits in females, Alt text: alanine aminotransferase; AST, aspartate aminotransferase; MS score, metabolic syndrome score.

The longitudinal relationships between MS score and serum characteristics in females were examined to help clarify the mechanism associated with MS in females. Additionally, we discovered that in females, ALT and hemoglobin had a positive correlation with MS score (*p* < 0.001 for both, Figure 3). Female AST or urea levels did not significantly correlate with the MS score.

### Bidirectional association of MS score with ALT in females

**Figure 4:**
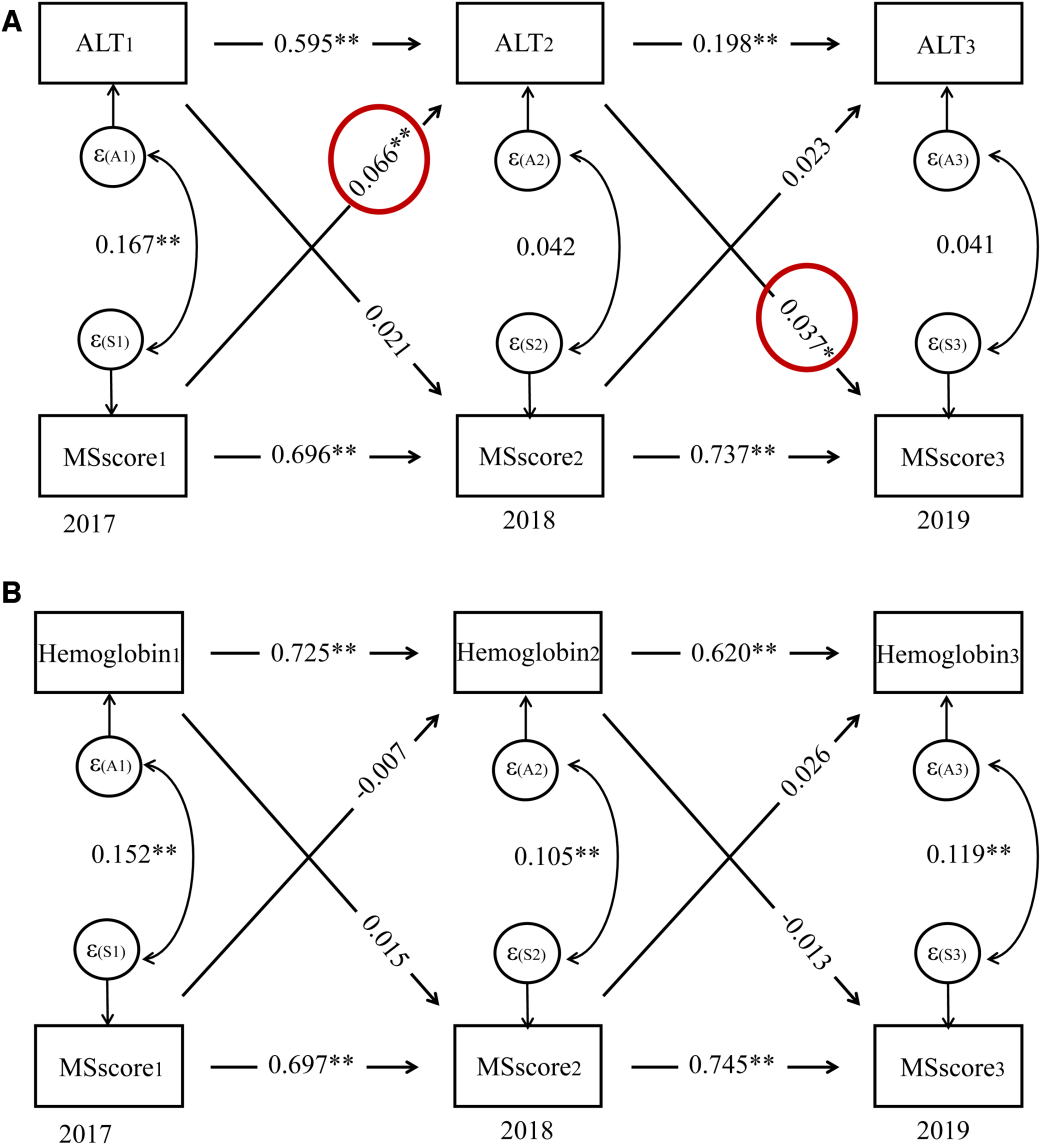
Cross-lagged panel model for serum traits and MS score in females, Alt text: alanine aminotransferase; MS score, metabolic syndrome score. *P < 0.05, **P < 0.01.

A similar pattern was seen between the repeated measures of ALT and hemoglobin (*p* < 0.001 for both), and the CLPM in females demonstrated that the MS score predicted the subsequent MS score at each time point (*p* < 0.001 for both). This auto-regressive part of the model showed that the temporal stability of ALT and hemoglobin from 2017 to 2019 were lower than the temporal stability of MS score over the same time period (ALT: β = 0.595 and 0.198, MS score: β = 0.696 and 0.737; hemoglobin: β = 0.725 and 0.620, MS score: β = 0.697 and 0.745). The CLPM also revealed that whereas ALT in 2017 did not predict the MS score in 2018, the MS score in 2017 did (β = 0.066, *p* = 0.003). ALT from 2018 did, however, foretell an MS score from 2019 (β = 0.037, *p* = 0.049). There was no bidirectional relation between hemoglobin and MS score (Figure 4).

### Sub-analysis of NAFLD's genetic variation

We also performed the SNPs test among 440 female participants in 2017 (NAFLD, *n* = 300; control, *n* = 140, Table 2), as genetic variables play crucial roles in the development and progression of NAFLD. Female NAFLD patients had significantly higher BMIs than the controls (*p* < 0.001). Table 3 provides more thorough details on the six SNPs.

**Table 2:** Participants information in the sub-analysis.

	NAFLD (*N* = 479)	Control (*N* = 253)	*P*
	Mean ± SD	Mean ± SD	
Female (%, *n*)	62.6%, 300	55.3%, 140	
Age (years)	72.14 ± 5.14	72.40 ± 5.27	0.882
BMI (kg/m^2^)	26.07 ± 3.00	23.55 ± 3.58	<0.001

BMI, body mass index; NAFLD, non-alcoholic fatty liver disease.

**Table 3:** The SNPs analyzed in this sub-analysis.

Gene	SNP ID	Chromosome	Function	Allele
BDNF	rs925946	11:27645655	Intron variant	G/T
LPL	rs15285	8:19967156	3 prime UTR variant	C/T
LPL	rs295	8:19958727	Intron variant	A/C
LPL	rs301	8:19959423	Intron variant	T/C
TCF7L2	rs7901695	10:112994329	Genic upstream transcript variant, intron variant	T/C
TCF7L2	rs7903146	10:112998590	Genic upstream transcript variant, intron variant	C/T

BDNF, Brain derived neurotrophic factor; LPL, Lipoprotein lipase; TCF7L2, Transcription factor 7 like 2; SNPs, single nucleotide polymorphisms.

HWE was met by all SNPs (*p* > 0.05). There was a significant difference between NAFLD and controls in the allele and genotype frequencies of rs15285, rs295, and rs301, as well as rs925946, rs7901695, and rs7903146 (Table 4).

**Table 4:** Allele and genotype distribution in the subjects of the sub-analysis.

SNPs	Allele frequency		X^2^	P	FDR	95% CI	Genotype frequency			X^2^	P	FDR	HWE
rs925946	G	T	3.914	0.047	0.289	0.409 [0.164∼1.019]	G/G	T/G		4.003	0.045	0.34	0.999
NAFLD	589 (0.984)	9 (0.015)					290 (0.969)	0 (0.030)					
Non-NAFLD	268 (0.964)	10 (0.035)					129 (0.928)	10 (0.071)					
rs15285	C	T	4.428	0.035	0.176	0.692 [0.491∼0.976]	C/T	T/T	C/C	4.698	0.095	0.357	0.999
NAFLD	488 (0.812)	108 (0.187)					90 (0.312)	9 (0.031)	189 (0.656)				
Non-NAFLD	207 (0.750)	69 (0.250)					51 (0.369)	9 (0.065)	78 (0.565)				
rs295	A	C	4.552	0.032	0.441	0.690 [0.490∼0.971]	A/C	A/A	C/C	4.906	0.086	0.425	0.999
NAFLD	487 (0.814)	111 (0.185)					93 (0.311)	197 (0.658)	9 (0.030)				
Non-NAFLD	209 (0.751)	69 (0.248)					51 (0.366)	79 (0.568)	9 (0.064)				
rs301	T	C	4.563	0.032	0.370	0.691 [0.492∼0.971]	T/C	C/C	T/T	4.633	0.098	0.473	0.999
NAFLD	486 (0.812)	112 (0.187)					92 (0.307)	10 (0.033)	197 (0.658)				
Non-NAFLD	210 (0.750)	70 (0.250)					52 (0.371)	9 (0.064)	79 (0.564)				
rs7901695	T	C	5.012	0.025	0.251	0.463 [0.232∼0.922]	T/T	T/C		5.229	0.022	0.222	0.902
NAFLD	559 (0.970)	17 (0.290)					271 (0.940)	17 (0.059)					
Non-NAFLD	259 (0.938)	17 (0.061)					121 (0.876)	17 (0.123)					
rs7903146	C	T	6.347	0.011	0.176	0.436 [0.225∼0.845]	C/C	T/C		6.648	0.009	0.148	0.999
NAFLD	558 (0.968)	18 (0.031)					270 (0.937)	18 (0.062)					
Non-NAFLD	257 (0.931)	19 (0.068)					119 (0.862)	19 (0.137)					

CI, confidence interval; FDR, false discovery rate; HWE, Hardy-Weinberg equilibrium; NAFLD, non-alcoholic fatty liver disease; SNPs, single nucleotide polymorphisms.

Since there was a strong longitudinal correlation between MS score and ALT in females, we also looked at the relationship between six potential NAFLD-related SNPs and MS score or ALT in females. In Table 5, the connection utilizing five genetic models is shown. The dominant model revealed a substantial correlation between the MS score and the *LPL* polymorphism rs295 (LPL). A higher MS score was statistically associated with the LPL rs295 AA genotype (*p* = 0.042). Rs295 and ALT were not shown to be significantly correlated. Supplementary Table S1 provides more details regarding associations between other SNPs and MS score/ALT. There were no statistically significant relationships between rs925946, rs15285, rs301, rs7901695, and rs7903146.

**Table 5:** Association between rs295 in *LPL* and MS score/ALT.

Association	Genotype	*N*	Mean difference [95% CI]	*P*
rs295-MS score	Codominant			
	A/A	179		0.121
	C/A	98	0.277 [0.011∼0.543]	
	C/C	14	0.186 [−0.402∼0.774]	
	Dominant			
	A/A	179		0.042
	C/A-C/C	112	0.265 [0.011∼0.52]	
	Recessive			
	A/A-C/A	277		0.768
	C/C	14	0.088 [−0.495∼0.671]	
	Over-dominant			
	A/A-C/C	193		0.501
	C/A	98	0.263 [0.001∼0.526]	
	log-Additive			
	0,1,2		0.196 [−0.017∼0.408]	0.072
rs295-ALT	Codominant			
	A/A	276	−1.119 [−3.381∼1.145]	0.329
	C/A	144	2.688 [−2.668∼8.043]	
	C/C	18		
	Dominant			
	A/A	276	−0.696 [−2.876∼1.485]	0.532
	C/A-C/C	162		
	Recessive			
	A/A-C/A	420	3.071 [−2.228∼8.369]	0.257
	C/C	18		
	Over-dominant			
	A/A-C/C	294	−1.283 [−3.522∼0.956]	0.262
	C/A	144		
	log-Additive		−0.127 [−1.977∼1.723]	
	0,1,2			0.893

ALT, alanine aminotransferase; CI, confidence interval; LPL, Lipoprotein lipase; MS score, metabolic syndrome score.

## Discussion

In this study, we found that only the WC grew with age in males, but both the WC and MS score did so in females. Additionally, we found substantial positive longitudinal relationships between ALT and hemoglobin and the MS score, which measures metabolic status and risk, in the senior Chinese female population. Additionally, it raises the possibility of causal relationships between the onset of MS and the rise of ALT in females caused by CLPM. A statistical link between *LPL* rs295 polymorphism and higher MS scores in females was also found.

Those with a BMI of 23.0–24.9 kg/m^2^ were classified as pre-obesity; females with a WC of 80 cm–85 cm and males with a WC of 85 cm–90 cm were classified as pre-obesity (27). In the present study, females in 2017 were pre-obese (BMI = 24.20 kg/m^2^, WC = 81.68 cm); their WC significantly increased with ageing. In comparison, males had no significant WC trend from 2017 to 2019. This may be due to the fact that men and women have different sex hormone levels, which may affect anthropometric measurements like WC. Seyfart et al. observed a negative association between WC and sex hormone-binding globulin only in women, so did De Sousa et al., who reported a positive association between WC and testosterone (28); while Svarberg et al. suggested that WC could predict testosterone levels in men. Another reason for the difference in WC between men and women might be the distinct metabolic profile (29). Men and women have varied patterns of body fat distribution. Research indicated a higher prevalence of visceral fat among men, whereas women tend to display larger subcutaneous fat accumulations (30) which may showed up in WC. As pre-obesity often occurs in parallel with MS, to further determine whether high-risk status in obesity-related measures has different associations with MS, we also checked for longitudinal interaction between serum measures, i.e., ALT, AST, hemoglobin, and urea related to MS score in females.

MS, a complex multifactorial disease, is a growing problem worldwide, especially in the elderly. We found a positive longitudinal association between hemoglobin and MS score. Hemoglobin concentration may reflect the functional iron in the body, which plays a vital role in the metabolic processes, e.g., immune function (31). Consistent with our finding, Nebeck et al. (32) showed a positive correlation between hemoglobin and MS in Ethiopia; According to Hashimoto et al. (33), greater hemoglobin levels were linked to an increased risk of MS in Japan; He et al. (34) observed that hemoglobin level was higher in participants with MS in China. In contrast, Timoteo et al. (35) reported that either high or low hemoglobin concentration led to T2D, whereas hemoglobin has a protective effect in the normal range.

In terms of elderly women, there was a positive longitudinal association between the MS score and ALT; besides, the MS score in 2017 predicted the ALT level in 2018 in females, and ALT in 2018 predicted the MS score in 2019 in this study using the repeated measurements in three different time points. This was similar to previous reports, which demonstrated that liver markers, e.g., ALT, AST, and gamma-glutamyl transferase (GGT), were biomarkers for chronic diseases, e.g., NAFLD, T2D, and MS (9, 17–19, 36–39). In a Japanese retrospective cross-sectional investigation, Miyake et al. (9) reported that ALT could be a useful indicator for the diagnosis of NAFLD; Vozarova et al. (18) observed that an increase in ALT level was associated with the degradation of insulin resistance and predicted the progression of T2D; Nakanishi et al. (38) found that GGT was a predictor for developing MS and T2D; Henley et al. (39) showed that ALT could predict the risk of MS in an epidemiological study. All these findings confirmed that NAFLD may be a feature of MS and explained the possible mechanisms that link ALT to MS. ALT is crucial because it can change the amino acid alanine into pyruvate, which takes part in the Krebs cycle, which generates cellular energy (40). With regard to the tricarboxylic acid cycle, ALT is quite important. This is a different name for the Krebs cycle. These all discuss the processes that result in adenosine triphosphate, the chemical that powers cells. Thus, it also provides a potential strategy in subjects with high MS scores to lower the ALT concentration to prevent the development of MS or its complication.

Evidence has shown that genetic susceptibility regulates the initiation and development of NAFLD. In this study, rs925946 in *BDNF*, rs15285, rs295, and rs301 in *LPL*, and rs7901695 and rs7903146 in *TCF7L2* were chosen based on prior knowledge pertaining to chronic diseases (21–23). A significant association between rs295 polymorphism in *LPL* and MS score was found in females. This was supported by previous studies. *LPL* influences metabolic dysfunction by catalyzing the rate-limiting step in absorbing and storing circulating triacylglycerol in adipose tissue, e.g., hepatic and visceral adipose tissues with ectopic fat deposits (41). It was reported that elderly Caucasian women with MS had lower LPL activity (42); there was a relationship between the rs3779788 variant in *LPL* and MS in Taiwanese adults (43); *LPL* variant rs271 was associated with coronary artery disease (CAD) in Europe (44); Malek et al. (45) demonstrated that rs295 in *LPL* may be indicative for CAD, and rs326 in *LPL* was associated with the increased risk of obesity in Kuwait. The cause of this varying association with MS-related traits or diseases may be ethnic and gender differences. Based on the findings mentioned above, our results further strengthened the evidence linking genetic variants in *LPL* (rs326) and MS.

The study has several benefits. First, a homogenous, regionally representative longitudinal study was conducted to investigate the bidirectional relationship between ALT concentration and the risk of MS. This study better adopted the temporal associations in this study than did logistic regression analysis, and it offered some suggestions for MS prevention, which might be avoided through monitoring and controlling ALT concentration. Second, this finding adds to the growing body of evidence supporting the involvement of *LPL* in the etiology of MS by linking the rs295 in *LPL* with MS score in old Chinese females. The study's main weakness is that hormones, such as estrogen, may have an impact on MS as it ages by influencing the longitudinal connection between MS score and ALT in females (46). Additionally, because genetic variations tend to be gender- or ethnic-specific, validation of various ethnic groups and regions among MS patients with larger sample sizes may be a better alternative to replication in another population. It has also been confirmed that the *LPL* variant rs295 may be a target for the diagnosis and treatment of MS. Notably, MS is a cluster of risk factors, including hypertension, hyperglycemia, dyslipidemia, and abdominal obesity; the risk of MS possibly varies due to the calculation formula of MS in different populations, and an alternative machine learning method to predict MS based on the specific population may be a more optimal method (47, 48).

## Conclusions

Our study among females suggests that an increase in ALT concentration and the risk of MS may have causal relationships. The SNP rs295 in LPL gene we identified has the potential to predict MS, one probable reason for the onset of MS and the rise of ALT in the senior Chinese Han population.

## Data Availability

The datasets presented in this study can be found: https://pan.baidu.com/s/1nG1qPYxREUF_FJhoBM2wOw, code: 1q9v.
