# Supplementary material for: A bidirectional link between metabolic syndrome and elevation in alanine aminotransferase in elderly female: a longitudinal community study
Source: Front Cardiovasc Med. 2023 Jun 20;10:1156123. doi: 10.3389/fcvm.2023.1156123 (PMC10318155; doi:10.3389/fcvm.2023.1156123)
Supplement: Supplementary file 1 [file Table1.docx]

**Supplementary Table 1** Genetic association between SNPs and serum traits in females (Continued…)

| **Association** | **Genotype** | **N** | **Mean difference [95%CI]** | **P** |  |
| --- | --- | --- | --- | --- | --- |
| rs925946-MS score | Codominant |  |  |  |  |
|  | G/G | 278 |  | 0.881 |  |
|  | G/T | 13 | 0.046 [-0.558~0.650] |  | |
|  | log-Additive |  |  |  | |
|  | 0,1,2 |  | 0.046 [-0.558~0.650] |  | |
| rs925946-ALT | Codominant |  |  |  | |
|  | G/G | 419 | 0.165 [-5.006~5.336] | 0.950 | |
|  | G/T | 19 |  |  | |
|  | log-Additive |  | 0.165 [-5.006~5.336] |  | |
|  | 0,1,2 |  |  |  | |
| rs15285-MS score | Codominant |  |  |  | |
|  | C/C | 174 |  | 0.214 | |
|  | C/T | 95 | 0.222 [-0.051~0.496] |  | |
|  | T/T | 14 | 0.305 [-0.291~0.900] |  | |
|  | Dominant |  |  |  | |
|  | C/C | 174 |  | 0.082 | |
|  | C/T-T/T | 109 | 0.233 [-0.029~0.494] |  | |
|  | Recessive |  |  |  | |
|  | C/C-C/T | 269 |  | 0.453 | |
|  | T/T | 14 | 0.226 [-0.363~0.815] |  | |
|  | Over-dominant |  |  |  | |
|  | C/C-T/T | 188 |  | 0.148 | |
|  | C/T | 95 | 0.200 [-0.070~0.470] |  | |
|  | log-Additive | 174 |  |  | |
|  | 0,1,2 | 95 | 0.191 [-0.026~0.408] | 0.086 | |
| s15285-ALT | Codominant |  |  |  | |
|  | C/C | 267 |  | 0.615 | |
|  | C/T | 141 | -1.120 [-3.439~1.200] |  | |
|  | T/T | 18 | 0.388 [-5.039~5.814] |  | |
|  | Dominant |  |  |  | |
|  | C/C | 267 |  | 0.405 | |
|  | C/T-T/T | 159 | -0.949 [-3.179~1.281] |  | |
|  | Recessive |  |  |  | |
|  | C/C-C/T | 408 |  | 0.777 | |
|  | T/T | 18 | 0.775 [-4.592~6.141] |  | |
|  | Over-dominant |  |  |  | |
|  | C/C-T/T | 285 |  | 0.329 | |
|  | C/T | 141 | -1.144 [-3.436~1.148] |  | |
|  | log-Additive | 267 |  |  | |
|  | 0,1,2 | 141 | -0.582 [-2.468~1.304] | 0.545 | |

ALT, alanine aminotransferase; CI, confidence interval; LPL, Lipoprotein lipase; MS score, metabolic syndrome score.

**Supplementary Table 1** (Continued)

| **Association** | **Genotype** | **N** | **Mean difference [95%CI]** | **P** |  |
| --- | --- | --- | --- | --- | --- |
| rs301-MS score | Codominant |  |  |  | |
|  | T/T | 179 |  | 0.153 | |
|  | C/T | 98 | 0.252 [-0.015~0.520] |  | |
|  | C/C | 16 | 0.255 [-0.299~0.810] |  | |
|  | Dominant |  |  |  | |
|  | T/T | 179 |  | 0.052 | |
|  | C/T-C/C | 114 | 0.253 [-0.002~0.507] |  | |
|  | Recessive |  |  |  | |
|  | T/T-C/T | 277 |  | 0.554 | |
|  | C/C | 16 | 0.166 [-0.383~0.715] |  | |
|  | Over-dominant |  |  |  | |
|  | T/T-C/C | 195 |  | 0.086 | |
|  | C/T | 98 | 0.231 [-0.032~0.495] |  | |
|  | log-Additive | 179 |  |  | |
|  | 0,1,2 | 98 | 0.193 [-0.015~0.401] | 0.070 | |
| rs301-ALT | Codominant |  |  |  | |
|  | T/T | 276 |  | 0.275 | |
|  | C/T | 144 | -1.257 [-3.520~1.006] |  | |
|  | C/C | 19 | 2.690 [-2.531~7.912] |  | |
|  | Dominant |  |  |  | |
|  | T/T | 276 |  | 0.473 | |
|  | C/T-C/C | 163 | -0.797 [-2.975~0.380] |  | |
|  | Recessive |  |  |  | |
|  | T/T-C/T | 420 |  | 0.237 | |
|  | C/C | 19 | 3.121 [-2.044~0.286] |  | |
|  | Over-dominant |  |  |  | |
|  | T/T-C/C | 295 |  | 0.211 | |
|  | C/T | 144 | -1.431 [-3.669~0.808] |  | |
|  | log-Additive | 276 |  |  | |
|  | 0,1,2 | 144 | -0.173 [-2.007~1.662] | 0.854 | |
| rs7901695-MS score | Codominant |  |  |  | |
|  | T/T | 261 |  | 0.871 | |
|  | T/C | 22 | -0.040 [-0.517~0.438] |  | |
|  | log-Additive |  |  |  | |
|  | 0,1,2 |  | -0.040 [-0.517~0.438] |  | |
| rs7901695-ALT | Codominant |  |  |  | |
|  | T/T | 392 | 1.307 [-2.675~5.289] | 0.520 | |
|  | T/C | 34 |  |  | |
|  | log-Additive |  | 1.307 [-2.675~5.289] |  | |
|  | 0,1,2 |  |  |  | |
| rs7903146-MS score | Codominant |  |  |  | |
|  | C/C | 259 | -0.020 [-0.479~0.439] | 0.932 | |
|  | C/T | 24 |  |  | |
|  | log-Additive |  | -0.020 [-0.479~0.439] |  | |
|  | 0,1,2 |  |  |  | |
| rs7903146-ALT | Codominant |  |  |  | |
|  | C/C | 389 | 1.380 [-2.452~5.211] | 0.481 | |
|  | C/T | 37 |  |  | |
|  | log-Additive |  | 1.380 [-2.452~5.211] |  | |
|  | 0,1,2 |  |  |  | |
